# Comprehensive genome data analysis of *Pseudomonas benzopyrenica* Ch9-16 Isolated from chili powder

**DOI:** 10.1016/j.dib.2025.111634

**Published:** 2025-05-12

**Authors:** Mayra Paola Mena Navarro, Merle Ariadna Espinosa Bernal, Ana Laura Vega Rodríguez, Daniel Alejandro Ferrusca Bernal, Juan Enrique de Jesús López, María Carlota García Gutiérrez, Karla Isabel Lira de León, Miguel Angel Ramos López, Aldo Amaro Reyes, José Alberto Rodríguez Morales, Héctor Pool, Carlos Guzmán Martínez, Erika Álvarez Hidalgo, Juan Campos Guillén

**Affiliations:** a Facultad de Química, Universidad Autónoma de Querétaro, Cerro de las Campanas S/N, Querétaro 76010, Mexico; b Center for Advanced Biomedical Research, School of Medicine, Autonomous University of Queretaro, Campus Aeropuerto Carretera a Chichimequillas S/N, Ejido Bolaños, 76140 Santiago de Querétaro, Qro., Mexico; c Facultad de Ingeniería, Universidad Autónoma de Querétaro, Cerro de las Campanas S/N, Querétaro 76010, Mexico

**Keywords:** *Pseudomonas benzopyrenica* Ch9–16, Complete genome, Antibiotic resistance genes (ARGs*)*, Virulence factor genes, Chili powder

## Abstract

We provide a comprehensive genome analysis of *Pseudomonas benzopyrenica* Ch9–16, a bacterial strain isolated from chili powder processed in Fresnillo, Zacatecas, Mexico. The genome was sequenced using the Illumina NovaSeq platform. Bioinformatic analyses, including assembly and annotation, were conducted on the BV-BRC platform. The genome, comprising 18 contigs and approximately 5.3 million base pairs with a GC content of 65.19 % and 4854 protein-coding sequences. Analysis identified multiple antibiotic resistance genes (ARGs) and virulence genes. This genome data serves as a valuable resource for understanding this bacterial specie and contributes to the database of microorganisms isolated from chili powder. The genome data was deposited at National Center for Biotechnology Information (NCBI) under accession number Bioproject ID PRJNA1062060, Bio Sample ID SAMN45486407. The genome accession number was JBLHDL000000000.

Specifications Table

**Table:** 

Subject	Biological sciences
Specific subject area	Microbiology, Genomics, Bioinformatics
Data format	Raw, Filtered and analyzed
Type of data	Complete genome sequence in FASTA format Table(s) Figure(s)
Data collection	*Pseudomonas benzopyrenica* Ch9–16 was isolated using MacConkey agar selective medium from chili powder processed at Fresnillo, Zacatecas, México. ZymoBIOMICS™DNA Miniprep Kit was used during DNA purification and sequenced with Illumina NovaSeq platform. For the bioinformatics analyses, the BV-BRC platform was used for adapter trimming, quality filtering, genome assembly, genome annotation, and phylogenetic tree construction; the Porksee platform was employed for the genome map, and the JSpeciesWS platform was used to assess species similarity via ANI. The AMR phenotype analysis revealed resistance to: ampicillin, cephalothin, cefotaxime, dicloxacillin, gentamicin, erythromycin, sulfamethoxazole/trimethoprim, penicillin, vancomycin, tetracycline, carbenicillin, chloramphenicol, and nitrofurantoin.
Data source location	Institution: Universidad Autónoma de Querétaro City/Town/Region: Querétaro, Qro. Country: México GPS coordinates: 20°35′28″N 100°24′36″O
Data accessibility	The assembly data is deposited in a public repository, and the analysed data are presented in this report. Repository name: *Pseudomonas benzopyrenica* Ch9–16 chromosome deposited in NCBI. Data identification number: JBLHDL000000000, Bio Project: PRJNA1062060, Bio Sample: SAMN45486407 Direct URL to data: https://www.ncbi.nlm.nih.gov/nuccore/JBLHDL000000000 https://www.ncbi.nlm.nih.gov/biosample/45486407 https://www.ncbi.nlm.nih.gov/bioproject/1062060

## 1 Value of the Data

• The complete genome sequence of *P. benzopyrenica* Ch9–16 could serve as a valuable resource for comparative genome studies with other environmental *Pseudomonas* species related.
• This data is valuable to chili powder producers due to offers insights into the genome of a bacterial isolate present in this foodstuff.
• The genome data is a valuable resource to comparative studies of virulence and antibiotic resistance genes with similar bacterial species, and its potential spread to other bacterial species.
• This data is useful during evaluation of microbial safety in chili powder.

## 2 Background

Pseudomonas benzopyrenica was proposed as a novel species within the genus Pseudomonas in 2023 [1]. The type strain BaP3 isolated from soil samples was highly efficient at degrading benzo(a)pyrene, a chemical class of polycyclic aromatic hydrocarbons considered pollutants and as mutagenic compounds [2]. Other related strain MLY92 was recovered from the leaf veins of a diseased tobacco plant [3]. Recently a bacteriome analysis on Mexican chili powder revealed a high diversity, with presence of beneficial and pathogenic microorganisms with presence of important antibiotic resistance genes [4]. In this respect, it is imperative to know whether bacteria isolated from chili powder have potential functions to be characterized. Therefore, analyzing genome sequencing data of bacterial strains isolated from chili powder can be essential to understand potential metabolic pathways, virulence and antibiotic resistance genes. Currently, there is few available genomic information of microorganisms isolated from chili powder. In this study, we present the genome analysis of *Pseudomonas benzopyrenica* Ch9–16 to provide valuable information and contribute to the knowledge of this bacterial specie.

## 3 Data Description

**Fig. 1:**
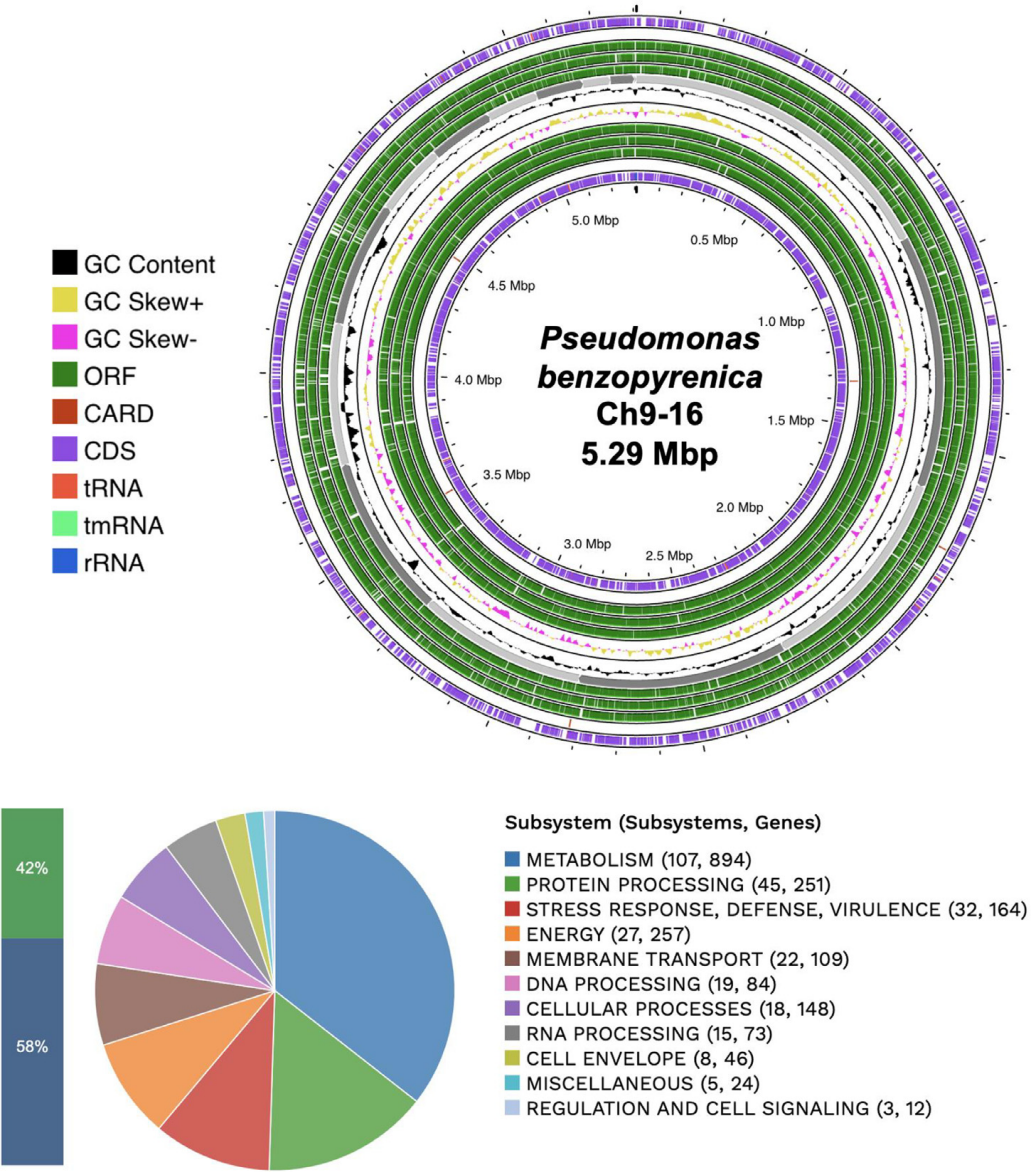
Circular genomic map and subsystems information for *P. benzopyrenica* Ch9–16**.** From the outermost layer to the center, the map includes assembled contigs, ORFs, CDS on the forward strand, CDS on the reverse strand, RNA genes, CDS similar to known antibiotic resistance genes, CDS similar to virulence factors, GC content, and GC skew. The distribution of subsystems is illustrated in the figure below. Subsystem coverage shows that 42 % corresponds to a total of 2062 genes, while 58 % represents genes not assigned to subsystems, totaling 2881 genes.

*P. benzopyrenica* Ch9–16 was isolated from chili powder sample elaborated in Fresnillo, Zacatecas, Mexico. Table 1 provides a summary of the genomic characteristics of *P. benzopyrenica* Ch9–16 obtained in BV-BRC platform [5]. The genome of the bacterium *P. benzopyrenica* Ch9–16 was submitted to the PATRIC Comprehensive Genome Analysis Service. According to the complete genome analysis of *P. benzopyrenica* Ch9–16, it contains 18 contigs with an estimated length of 5292,684 bp and an average guanine-cytosine content of 65.19 %. The N50 length, which is defined as the shortest sequence length at 50 % of the genome, is 516,804 bp. The CheckM Completeness is 95.1 % and CheckM Contamination is 4.5 %. Additionally, the genome was annotated using the RAST tool (Rapid Annotation using Subsystem Technology), and a unique genome identifier number, 237,610.49, was assigned to it. Based on the data provided by RASTtk, the genome comprises 4854 protein-coding sequences (CDS), 59 transfer RNA (tRNA) genes, and 3 ribosomal RNA (rRNA) genes. Virulence factors, antibiotic resistance genes, transporter genes, and drug target genes are detailed for each source. Additionally, the distribution of subsystems is described, representing key biological and metabolic processes (Fig. 1).

**Table 1:** Genomic description of *P. benzopyrenica Ch9–16*.

Characteristics	Source	Total
Genome Length	PATRIC	5292,684 bp
Number of contigs	PATRIC	18
Number of proteins characterized	PATRIC	3695
Number of putative/hypothetical proteins	PATRIC	1159
Number of rRNA genes	PATRIC	3
Number of tRNA genes	PATRIC	59
Number of proteins with pathway annotation	KEGG	878
*G* + *C* %	PATRIC	65.19 %
N50 contig size (bp)	PATRIC	516,804
Virulence factors	Victors	17
Virulence factors	VFDB	20
Virulence factors	PATRIC_VF	1
Antibiotic resistance genes	CARD	4
Antibiotic resistance genes	PATRIC	50
Transporter genes	TCDB	30
Drug target genes	DrugBank	15
Drug target genes	TTD	4

**Fig. 2:**
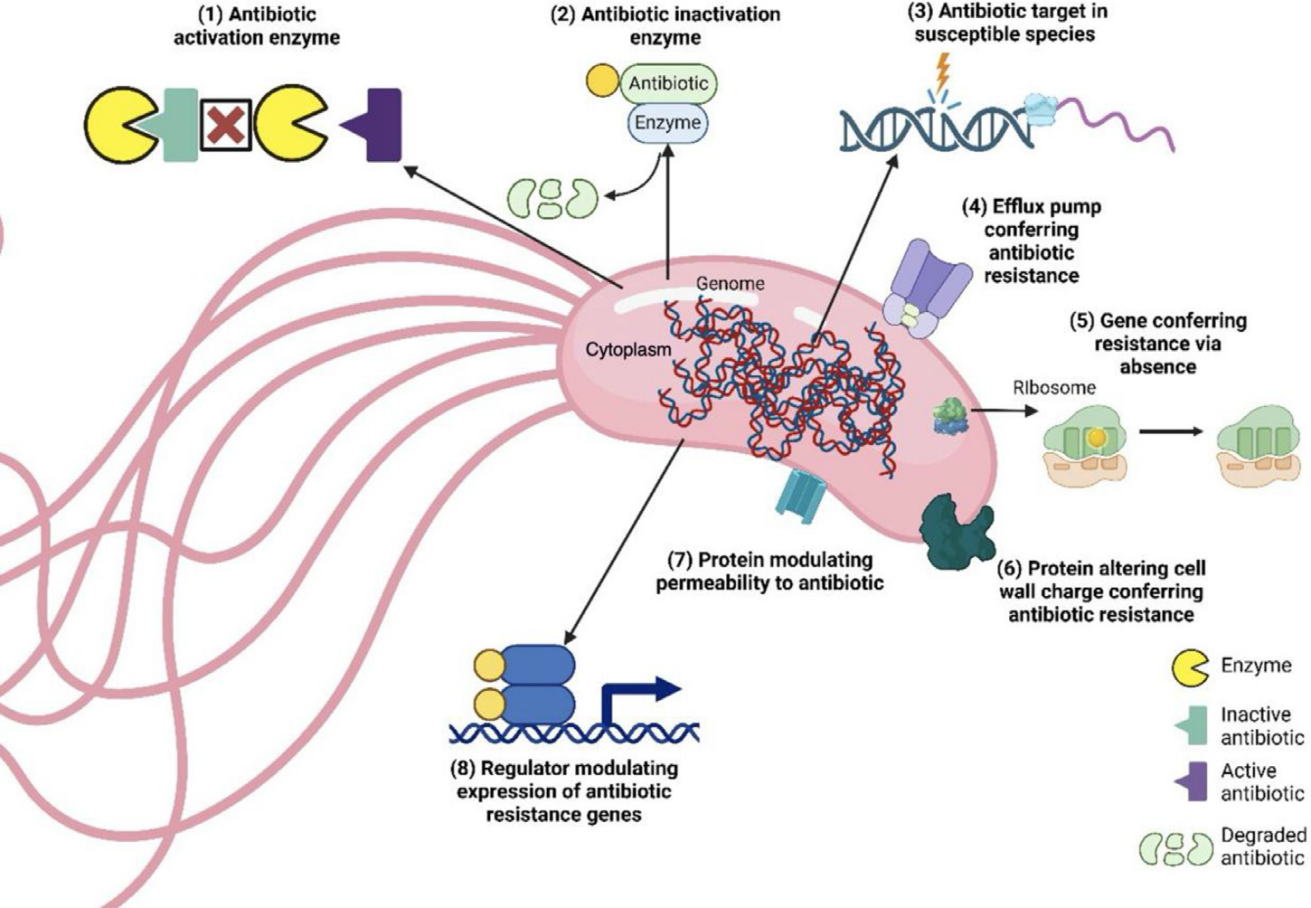
Mechanisms of antibiotic resistance predicted in *P. benzopyrenica* Ch9–16. (1) antibiotic activation enzyme, (2) antibiotic inactivation enzyme, (3) antibiotic target in susceptible species, (4) efflux pump conferring antibiotic resistance, (5) gene conferring resistance via absence, (6) protein altering cell wall charge conferring antibiotic resistance, (7) protein modulating permeability to antibiotic and (8) regulator modulating expression of antibiotic resistance genes. Created using Biorender under an academic license.

Fig. 2 presents the predicted antibiotic resistance genes identified through data source analysis, categorized into eight mechanisms of action. The mechanisms are as follows: (1) Antibiotic activation enzyme, represented by the katG gene; (2) Antibiotic inactivation enzyme, represented by the PDC family; (3) Antibiotic target modification in susceptible species, represented by alr, ddl, dxr, ef-G, ef-Tu, folA, dfr, folP, gyrA, gyrB, inhA, fabI, iso*tRNA*, kasA, murA, rho, rpoB, rpoC, s10p, and s12p genes; (4) Efflux pumps conferring antibiotic resistance, represented by emrAB-OMF, emrAB-TolC, mdtABC—OMF, mdtABC-TolC, mexAB-OprM, mexEF-OprN, mexEF-OprN system, and triABC-opmH genes; (5) Genes conferring resistance through absence, represented by the gidB gene; (6) Proteins altering cell wall charge to confer resistance, represented by gdpD and pgsA genes; (7) Proteins modulating permeability to antibiotics, represented by occD1/OprD, occD3/OpdP, occD7/OpdB, occK11/OpdR, oprB, oprD family, and oprF genes; and (8) Regulators modulating the expression of antibiotic resistance genes, represented by the oxyR gene*.*

Based on the bacterial virulence factors (VDFB) source, the virulence-related genes are shown in the Table 2.

**Table 2:** Predicted virulence-related genes in *P. benzopyrenica* Ch9–16 identified through VFDB analysis.

Gen	Product	Mechanism of action
*waaG*	UDP-glucose:(heptosyl)LPS alpha1,3-glucosyltransferase WaaG	Adhesion, Endotoxin
*waaF*	ADP-heptose–lipooligosaccharide heptosyltransferase II	
*fliI*	Flagellum-specific ATP synthase FliI	Adherence, Motility
*fliG*	Flagellar motor switch protein FliG	
*flhA*	Flagellar biosynthesis protein FlhA	
*fliM*	Flagellar motor switch protein FliM	
*flgG*	Flagellar basal body rod protein FlgG	
*fliP*	Flagellar biosynthesis protein FlipP	
*fliN*	Flagellar motor switch protein FliN	
*fleN*	Flagellar synthesis regulator FleN	
*motA*	Flagellar motor rotation protein MotA	
*pilH*	PilH twitching motility protein	Adhesion, Spasmodic motility.
*pilT*	PilT twitching motility protein	
*pilG*	PilG twitching motility protein	
*pilU*	Type IV pilus assembly ATPase component PilU	
*pilM*	Type IV pilus biogenesis protein PilM	
*algB*	Response regulator of the two-component system of alginate biosynthesis AlgB	Antiphagocytosis, Serum resistance
*algU*	RNA polymerase sigma factor RpoE	
*algR*	AlgR, a two-component response regulator of alginate biosynthesis system	
*algC*	Phosphoglucomutase (EC 5.4.2.2) @ Phosphomannomutase (EC 5.4.2.8)	

**Fig. 3:**
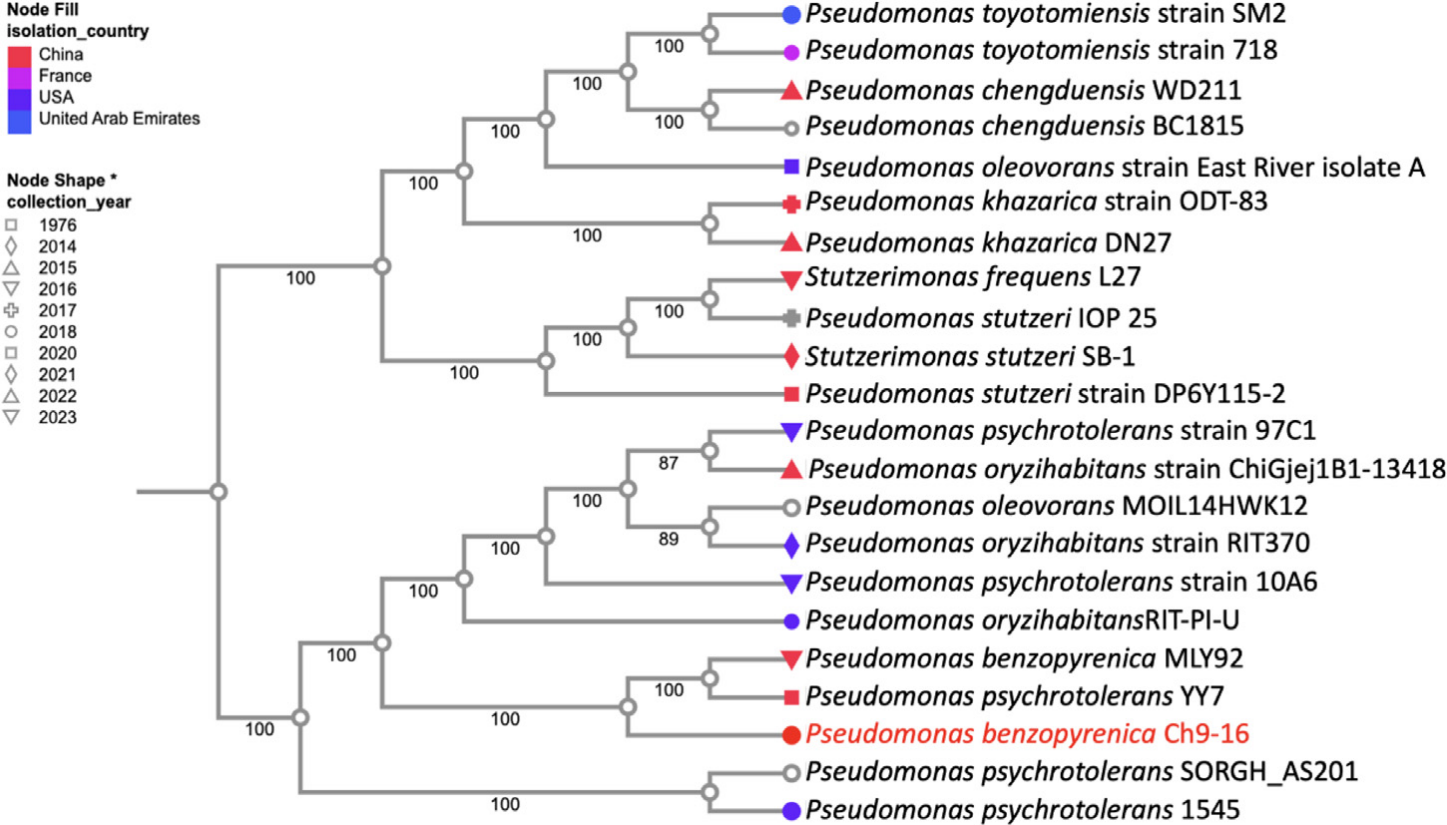
Phylogenetic tree including different species of *Pseudomonas*. The following strains were included in the codon tree pipeline analysis on BV-BRC platform: *Pseudomonas toyotomiensis* strain SM2 (554,344.14), *Pseudomonas toyotomiensis* strain 718 (554,344.13), *Pseudomonas chengduensis* WD211 (489,632.42), *Pseudomonas chengduensis* BC1815 (489,632.30), *Pseudomonas oleovorans* strain East River isolate A (301.32), *Pseudomonas khazarica* strain ODT-83 (2502,979.4), *Pseudomonas khazarica* DN27 (2502,979.8 ), *Stutzerimonas frequens* L27 (2968,969.19), *Pseudomonas stutzeri* IOP 25 (316.648)*, Stutzerimonas stutzeri* SB-1 (316.744)*, Pseudomonas stutzeri* strain DP6Y115–2 (316.633)*, Pseudomonas psychrotolerans* strain 97C1 (237,610.33)*, Pseudomonas oryzihabitans* strain ChiGjej1B1–13,418 (47,885.46)*, Pseudomonas oleovorans* MOIL14HWK12 (1033,992.3)*, Pseudomonas oryzihabitans* strain RIT370 (47,885.6)*, Pseudomonas psychrotolerans* strain 10A6 (237,610.35)*, Pseudomonas oryzihabitans* RIT-PI-U (47,885.57)*, Pseudomonas benzopyrenica* MLY92 (2993,566.4)*, Pseudomonas psychrotolerans* YY7 (237,610.37)*, Pseudomonas psychrotolerans* SORGH_AS201 (237,610.41) *and Pseudomonas psychrotolerans* 1545 (237,610.42). The color and shape of each node represent the country and year of collection for each isolated strain.

The phylogenetic tree of *P. benzopyrenica Ch9–16* is shown in Fig. 3. The data showed that Ch9–16 is closely related to *Pseudomonas benzopyrenica* MLY92 that was isolated of diseased tobacco leaves and *Pseudomonas psychrotolerans* YY7 that was isolated of plant.

The ANI values supported the phylogenetic analyses (Table 3). A pairwise genome comparison of P. benzopyrenica Ch9–16 revealed ANI values of 97.24 % with P. benzopyrenica BaP3, confirming its classification as P. benzopyrenica.

**Table 3:** Paired analysis of Average Nucleotide Identity (ANI) among *Pseudomonas* species. ANI values ≥ 95 % are highlighted in red.

The analysis of the AMR phenotype revealed resistance to: ampicillin, cephalothin, cefotaxime, dicloxacillin, gentamicin, erythromycin, sulfamethoxazole/trimethoprim, Ciprofloxacin, Clindamycin, penicillin, vancomycin, tetracycline, carbenicillin, chloramphenicol, and nitrofurantoin. The bacterial strain showed sensitive to antibiotics: amikacin, netilmicin, imipenem, tobramycin, cefepime, ceftazidime, ceftriaxone, levofloxacin, meropenem, piperacillin-tazobactam and norfloxacin (Table 4).

**Table 4:** Antibiotic susceptibility of *P. benzopyrenica* Ch9–1*6.*

Antibiotic	Phenotype	Antibiotic	Phenotype	Antibiotic	Phenotype
Ampicillin (10 µg)	R	Ciprofloxacin (5 µg)	R	Erythromycin (15 µg)	R
Cephalothin (30 µg)	R	Clindamycin (30 µg)	R	Gentamicin (10 µg)	R
Cefotaxime (30 µg)	R	Dicloxacillin (1 µg)	R	Penicillin (6 µg)	R
Tetracycline (30 µg)	R	Amikacin (30 µg)	S	Netilmicin (30 µg)	S
Sulfamethoxazole/ Trimethoprim (25 µg)	R	Carbenicillin (100 µg)	R	Nitrofurantoin (300 µg)	R
Vancomycin (30 µg)	R	Chloramphenicol (30 µg)	R	Norfloxacin (10 µg)	S
Levofloxacin (2 µg)	S	Cefepime (4 µg)	S	Imipenem (1 µg)	S
Meropenem (1 µg)	S	Ceftazidime (1 µg)	S	Tobramycin (4 µg)	S
Piperacillin-Tazobactam	S	Ceftriaxone (1 µg)	S		

*S*=Sensitive; *R*=Resistant.

The results of the biochemical analysis are presented in Table 5.

**Table 5:** Biochemical properties of *P. benzopyrenica* Ch9–1*6.*

Biochemical Test	Result	Biochemical Test	Result
Glucose	Positive	Urea	Negative
Sucrose	Negative	Lysine	Negative
Sorbitol	Positive	Esculin	Negative
Rhamnose	Negative	Voges-Proskauer	Negative
Inositol	Positive	Citrate	Positive
Adenosine	Negative	Maltose	Positive
Melibiose	Negative	Raffinose	Negative
TSI (H_2_S)	Negative	Indole	Negative
Arginine	Negative	Tartrate	Positive
Ornithine	Negative	Acetamide	Positive

## 4 Experimental Design, Materials and Methods

### 4.1 Sample collection and microbial isolation

The chili powder sample from Fresnillo, Zacatecas was processed to isolate bacterial colonies using MacConkey agar, followed by incubation at 37 °C for 24 hours. Based on their phenotype and resistance to ampicillin (100 µg/mL) on tryptic soy agar (TSA) medium (Difco Laboratories, Detroit, MI, USA) the strain Ch9–16 was selected for further characterization.

### 4.2 Identification by microscan panel

Biochemical tests were conducted following standard procedures [6]. The bacterial culture was obtained (1 × 10⁶ CFU/mL) and prepared using the Prompt™ system and distributed into wells of a Neg Combo Panel Type 68 using the MicroScan Renok (Beckman Coulter, USA). Mineral oil was added to specific wells, and the panels were incubated at 35 °C for 24 hours under aerobic conditions. After adding detection reagents, the panels were analyzed with the MicroScan AutoSCAN-4 system. The LabPro software identified the genus, species, and microbial susceptibility with probabilities of ≥85 %.

### 4.3 Genome sequencing, assembly and annotation

Genomic DNA from P. benzopyrenica Ch9–16 was extracted using the ZymoBIOMICS™ DNA Miniprep Kit and sequenced at Zymo Research with Illumina® technology. Bioinformatics analysis was performed at BV-BRC using services such as Fastq Utilities, PATRIC, and RASTtk for genome annotation [5,[7], [8], [9], [10]]. Reference genomes were identified with Mash/MinHash, protein families aligned with MUSCLE v5, and phylogenetic analysis conducted with RaxML. A circular genome map was generated using Proksee [11,12]; while species similarity was assessed with JSpeciesWS through ANI [13]. Phylogenetic trees were created using the Codon Tree pipeline from BV-BRC, such as strain *Pseudomonas toyotomiensis* strain SM2 (554,344.14), *Pseudomonas toyotomiensis* strain 718 (554,344.13), *Pseudomonas chengduensis* WD211 (489,632.42), *Pseudomonas chengduensis* BC1815 (489,632.30), *Pseudomonas oleovorans* strain East River isolate A (301.32), *Pseudomonas khazarica* strain ODT-83 (2502,979.4), *Pseudomonas khazarica* DN27 (2502,979.8 ), *Stutzerimonas frequens* L27 (2968,969.19), *Pseudomonas stutzeri* IOP 25 (316.648)*, Stutzerimonas stutzeri* SB-1 (316.744)*, Pseudomonas stutzeri* strain DP6Y115–2 (316.633)*, Pseudomonas psychrotolerans* strain 97C1 (237,610.33)*, Pseudomonas oryzihabitans* strain ChiGjej1B1–13,418 (47,885.46)*, Pseudomonas oleovorans* MOIL14HWK12 (1033,992.3)*, Pseudomonas oryzihabitans* strain RIT370 (47,885.6)*, Pseudomonas psychrotolerans* strain 10A6 (237,610.35)*, Pseudomonas oryzihabitans* RIT-PI-U (47,885.57)*, Pseudomonas benzopyrenica* MLY92 (2993,566.4)*, Pseudomonas psychrotolerans* YY7 (237,610.37)*, Pseudomonas psychrotolerans* SORGH_AS201 (237,610.41) *and Pseudomonas psychrotolerans* 1545 (237,610.42). DNA sequences were deposited in NCBI under BioProject ID PRJNA1062060 with the genome accession number JBLHDL000000000.

### 4.4 Antibiotic sensitivity test

Antibiotic resistance and sensitivity in P. benzopyrenica Ch9–16 were assessed using microdilution with a MicroScan system and disk diffusion, following CLSI guidelines [14]. Antibiotic discs (Oxoid) were employed with ampicillin (10 µg), carbenicillin (100 µg), cephalothin (30 µg), cefotaxime (30 µg), ciprofloxacin (5 µg), clindamycin (30 µg), dicloxacillin (1 µg), erythromycin (15 µg), penicillin (10 U), tetracycline (30 µg), vancomycin (30 µg), amikacin (30 µg), chloramphenicol (30 µg), gentamicin (10 µg), netilmicin (30 µg), nitrofurantoin (300 µg), norfloxacin (10 µg), sulfamethoxazole/trimethoprim (25 µg), cefazolin (2 µg/mL), levofloxacin (2 µg/mL), meropenem (1 µg/mL), cefepime (4 µg/mL), ceftazidime (1 µg/mL), ceftriaxone (1 µg/mL), tigecycline (2 µg/mL), cefuroxime (4 µg/mL), ertapenem (0.5 µg/mL), imipenem (1 µg/mL), and tobramycin (4 µg/mL).

## Limitations

Not applicable.

## Ethics Statement

This work does not involve human subjects or animal subjects. The authors declare that this manuscript is original work and has not been published elsewhere.

## CRedit Author Statement

**Mayra Paola Mena Navarro:** Writing-original draft; **Merle Ariadna Espinosa Bernal:** Conceptualization, Methodology; **Ana Laura Vega Rodríguez:** Writing- original draft, Methodology; **Daniel Alejandro Ferrusca Bernal** Software, Data curation; **Juan Enrique de Jesús López:** Software, Data curation; **Maria Carlota García Gutiérrez:** Writing-original draft, Methodology; **Karla Isabel Lira De León:** Writing- original draft, Resources, Methodology; **Miguel Angel Ramos Lopez:** Writing-review & editing; **Aldo Amaro Reyes:** Writing-review & editing; **José Alberto Rodríguez Morales:** Writing-review & editing; **Héctor Pool:** Resources, Writing-review & editing; **Carlos Guzmán Martínez:** Writing-review & editing; **Erika Álvarez Hidalgo**; Conceptualization, Methodology; **Juan Campos Guillen:** Validation, Supervision, Resources, Writing-review & editing, Supervision.

## Data Availability

National Center for Biotechnology InformationPseudomonas benzopyrenica (Original data).
